# The Importance of the Microscope in the Diagnosis of Disease

**Published:** 1889-10

**Authors:** E. W. Mulligan

**Affiliations:** 290 West Avenue; Rochester, N. Y.


### THE IMPORTANCE OF THE MICROSCOPE IN THE DIAGNOSIS OF DISEASE.[1]

By E. W. MULLIGAN, M. D., Rochester, N. Y.

I PRESUME that you will all agree with me when I say that to make a correct diagnosis is the most difficult part of our work. A diagnosis once made, the treatment is comparatively easy. It is when we are not sure of our diagnosis that we find the treatment so troublesome. In very many cases the microscope is our best consultant. You must have a first-class instrument with at least two objectives—much better to have three or four. And as good light is of so much importance, you should have an Abbé condenser, or a Bausch and Lomb's sub-stage condenser. It requires much time and practice to become expert with the microscope; but it is time well spent. In the examination of urine it is called into use every day by the busy practitioner. How is it possible to make an exact diagnosis of renal or vesical disease without the aid of the microscope?

Take a case of chronic interstitial nephritis in its incipient stage, when you have no symptom or sign that points directly to the kidneys; simply, indefinitely, out of health or suffering with dyspepsia. You may examine the urine for albumen and find none; or, if you do find it, it is but a trace. In such a case the microscope is our best friend and safest consultant, and, if we understand well how to interrogate it, it will certainly make out the diagnosis for us. But, never make a positive diagnosis on a single examination. You may find a small hyaline cast or two, and yet no disease of the kidneys be present. You may not find casts at all, and yet disease exist. It is necessary to examine the sediment of the whole twenty-four hours' urine, and examine several times in many cases before pronouncing your verdict. This is a very important point. I have known very great injustice to be done to a patient by the physician making a diagnosis of Bright's disease after finding one or two small hyaline casts and a trace of albumen. It is a very serious matter to give a grave prognosis to your patient, or to your patient's family, even when you tell them the exact truth. But to give a grave prognosis after finding a little albumen present at a single examination, is a great mistake. The careful use of the microscope will eventually clear up all doubts in our minds regarding almost any case of renal disease. If you find a trace of albumen in a urine, how are you to know whether the albumen comes from a diseased kidney, or from the bladder, *or not* from either. If you do not call upon your microscope, you will certainly be at sea.

1. Read before the Rochester Pathological Society, June 20, 1889.

If your patient be a female, it will be necessary to exclude the possibility of leucorrheal discharge. The finding of large pavement epithelium, together with pus cells, is pretty sure evidence of this condition being present, but not so positive as catheterizing your patient and examining the specimen thus taken.

Many cases of gravel are passed by, by the physician, without a thorough understanding of the case. It is not necessary to have large calculi passing through the ureter in order to produce severe paroxysms of pain. The passage of uric acid in large quantities produces the "uric acid storm." The examination of the patient's urine from day to day, and finding large quantities of these crystals, will make the diagnosis positive. I should like to add more concerning the examination of urine, but this is so large a subject that we will not have time in this short paper to more than mention the few points given.

Regarding tuberculosis, we affirm (with excellent authority) that it is curable if discovered and properly cared for in time. "We know, now, that tubercle, wherever it is found in the bones, in the glands, in the testicle, in serous membranes, as well as in the lungs, may be transformed into fibrous tubercle, and this tuberculosis localized elsewhere, having become sclerous, gives no more sign of its presence during the existence of the patient." "Grancher and Renault show that whatever be its form, tubercle always contains a fibrous zone in miniature, which, if the development of the tubercle is slow or arrested, may increase and lead to a natural cure." In the beginning stage of phthisis, a certainty in diagnosis can only be acquired by the micro-chemical method. To stain the bacilli of tuberculosis, I have found the old method of Ehrlich to be the most satisfactory. Of course, this is slow and requires about twenty-four hours to complete the examination. An easier and more rapid method is that of Ziehl. By this method we can ascertain if tubercle bacilli be present in a specimen in about fifteen minutes. I received the formula from Dr. Roseboom, who brought it with him from Vienna. It is—

| | |
|---|---|
| Carbolic acid, | 5.00 |
| Alcohol, | 10.00 |
| Distilled water, | 100.00 |
| Fuchsin, | 1.00 |

To decolorize, use twenty-five per cent. sulphuric acid.

Local tuberculosis may become infectious unless we remove the source from which the bacilli come. We can only ascertain positively that it is tuberculosis by making the micro-chemical test. The diagnosis being made, the offending organ or gland should, if practicable, be removed, as the lymphatic vessels are the principal means of transport for the infection, and general tuberculosis may be the result unless this is done.

Austin Flint taught that phthisis was never primative in the larynx. It was Virchow who first made this subject understood. Frantzel has described in ten cases of laryngitis, the importance of tubercle bacilli in the sputum which is expectorated, or taken from the larynx by the aid of the brush. This I have done, and have proven that phthisis is primative in the larynx.

Supposing we have a case of pharyngeal ulcer, the differential diagnosis is easy. Scrape a little mucus or pus from the surface of the ulcer, examine this for Koch's bacillus, and if none be present you have not tuberculosis or scrofulosis. Right here let me add that scrofula is peripheral, while tuberculosis is central.

They are both produced by the same cause. Wherever you find the bacilli of Koch, you have phthisis. This disease, although presenting itself in many different forms, is always the same bacillosis and produced by the same cause, the tubercle bacillus. As Germain See says, "There are not six species of phthisis, as Boyle wished to establish, nor two—tubercular and inflammatory. It is always tubercular as taught by Laënnec, and always bacillary as demonstrated by Koch. To demonstrate to you the great value of ascertaining whether or not the germ of consumption be present, permit me to relate a case. Mrs. M. had been very ill for several weeks. Both lungs were in the condition you might reasonably expect to find a case of miliary tuberculosis. Respirations and pulse were rapid, temperature went up as high as 108° F. A consultation was had and a prognosis of death given. The physician was discouraged and so were the family. It was about this time that I saw her with her physician. We examined the sputum, and much to our surprise found *no* bacilli. We examined again and still did not find evidence of tuberculosis. This was encouraging. We now concluded that it was (as her physician diagnosed at the beginning) catarrhal pneumonia, and that if we could keep her alive by the aid of stimulants for a few days until the lungs should begin to clear up, she might get entirely well. We gave a favorable prognosis. The family were encouraged, the physicians were encouraged, and the patient partook of the same,—for we told her that she would get well,—and she did recover entirely, and to-day is the picture of good health. How important was the negative result of the micro-chemical examination of this sputum? To prove to you that phthisis is curable, I must tell you that the records of the autopsies at the New York City Hospital show that more than one-half of those on whom *post mortems* have been made have had consumption. I have had considerable experience in making autopsies myself, and in fully one-half of all cases examined I have found old scars and other evidences of cured phthisis. Prof. Bronardel in medico-legal autopsies on individuals reputed to be healthy found four-fifths with tubercular lesions in all stages.

In growths of the nose and throat it is often necessary to know the nature of the growth in order to determine whether to operate or not. This can be done very easily, by cutting a small piece off of the growth, staining, and examining with microscope. This makes the diagnosis and the treatment and prognosis come easily after that.

Many times we see cases of "leucorrhea," so-called. We cannot get a history of gonorrheal infection, yet we half suspect that it is specific. How are we to prove our suspicions or make a diagnosis? By the aid of the microscope.

Wm. Jap. Sinclair says: "All experience goes to show that the presence of the gonococcus settles the question; it is the pathognomonic sign of the disease. If gonococci are present in the discharge from an inflamed mucous membrane, the discharge is of gonorrheal origin." Thus you see the method of making a positive diagnosis is easy.

When we have a case of ophthalmia in a new-born child, we should never fail to look for the "gonococcus Neisser." If this were done oftener, many children could be saved from being totally blind. I have known of a physician, when his attention was called to the eyes of a new-born child, say, "Oh, just put a little breast milk into the baby's eyes and they will be all right," and the result was the child lost the sight of both eyes.

The discovery of the tubercle bacillus by Koch was but the beginning of the discovery of the causes of many diseases hitherto shrouded in mystery. It was years before all or a majority accepted this as a truth. Now it is universally accepted.

Another great discovery is the finding of the "hematozoa of malaria" or the "oscillaria malariæ" of Laveran. The word malaria is too frequently used to hide our ignorance. The term is used so loosely that when we say to a patient, "You are afflicted with malaria," he is as wise as before we spoke. The time has, I believe, come when we need no longer speak of malaria without a definite understanding of what we mean by that word. Prof. Welch, of Johns Hopkins University, tells me that he considers that the microörganism of malaria is as *surely* present in the blood of patients suffering with intermittent fever as the bacillus of Koch is present in consumption. This organism—

not a bacillus—was first described by Laveran and communicated to the Paris Academy of Medicine in 1881 and 1882. He found in the blood of persons attacked with malaria: first, crescentic pigmented bodies; second, pigmented bodies in the interior of red blood corpuscles which underwent changes in form described as amæboid; and third, a pigmented flagellate organism. These observations were confirmed by Richard, Marchiafava and Celli, and also Councilman and Osler, of Baltimore.

It is an easy matter to get the blood. But, first, you must be certain to cleanse the finger, or whatever portion of the body you take it from, before introducing your needle. This is important, as the skin often contains coal dust or some pigment that will interfere with your microscopical examination. Examine the blood fresh without any other preparation.

Klebs and Tommasi-Crudeli claimed to have discovered the *bacillus malariæ* some two years before Laveran described the microörganisms referred to; but Laveran and those who confirm his discovery do not even mention the bacillus discovered by Klebs, although they must have known of this supposed discovery of the bacillus.

Prof. Osler, to whose paper I am indebted for most of my knowledge concerning the hematozoa of malaria, examined the blood of seventy patients suffering with malaria and found these organisms in all except eight, and nearly all of these eight had taken quinia. Quinia causes the hematozoa to disappear. I have seen these organisms but once, and it was in a typical case of intermittent fever. I believe that if we examine the blood of all cases of doubtful malaria, that we will convince ourselves that this disease is not as prevalent as we have been taught to believe.

A certain physician said to me not many months ago: "Doctor, I tell you that quinine in big doses will cure cases of anemia when other remedies fail." I think I can see now why this is true. The microörganism of malaria destroys the hemoglobin of the red blood corpuscle, and of course produces anemia, and in these cases of chronic malarial anemia all ordinary remedies used for anemia fail because they do not destroy the hematozoa of malaria. Your large doses of quinia accomplish this, and then your patient is in a position to improve with the aid of good nourishment, etc. I hope to be able to demonstrate to you the presence of these pigmented bodies in the blood of malarial subjects before very long. It is necessary to examine the blood at or about the time of the chill, unless it be a case of chronic malaria where there is no distinct chill. When I was a student at Bellevue, Professor Flint, now dead, taught that there was such a disease as "typho-malaria." This is now proven to be false. To prove that a given case is not malaria, but *enteric fever*, all you have to do is to examine the blood—a negative result would satisfy me that it was not malaria. Since writing what I have on malaria, I have received the first volume of the Cyclopedia of Diseases of Children, edited by Keating. I find that in this work Forchheimer has written a chapter on malaria, and has inserted some of the figures shown by Osler, showing the pigmented bodies in the red blood corpuscles. He also says regarding the value of the discovery of this microörganism:

> Cases have been reported in which the absence of the plasmodium in the blood was sufficient to disestablish a diagnosis, and in my own experience two cases have occurred which have conclusively proved to me the importance of examining the blood in doubtful malarial cases. The one case was an atypical typhoid fever, in which, as there was a strong tendency to an intermittent type of fever, the diagnosis of intermittent fever had been made and corrected by the repeated failure to find the plasmodium, and in which the patient finally died of a perforation of the intestines. The typhoid lesions and perforations were all demonstrated by autopsy. The other case was one in which the diagnosis of typhoid had been made, and in which the plasmodium was found during a chill and promptly relieved hy a large dose of quinine. Not only in grave cases like these, but also in comparatively mild ones, neuralgia, intestinal troubles, etc., the plasmodium, or hematozoa, can be demonstrated, and with patient search, a *positive diagnosis* can always be arrived at.

Now, gentlemen, I think I have demonstrated to you the great value of the microscope in assisting us in making diagnoses. To be sure, I have mentioned but a few diseases in which it is of great service to us as practitioners; but I think that I can safely say that in every case of prolonged illness the microscope should be called into service, either for the examination of the sputum, the urine, or some other secretion or excretion.

I am very thankful to you, gentlemen, for the honor conferred upon me the past year.

290 West Avenue.

